# Clinical Validation of the QMAC-DST System for Testing the Drug Susceptibility of *Mycobacterium tuberculosis* to First- and Second-Line Drugs

**DOI:** 10.3389/fmicb.2019.00706

**Published:** 2019-04-16

**Authors:** Sangyeop Lee, Daehyun Chu, Youn Mi Choi, EunJi Jo, Suyeoun Kim, Haeun Kim, Hyun Jung Kim, Jeonghyun Chang, Heungsup Sung, Geumrae Kang, Bonghwan Jin, Eun-Geun Kim, Sunghoon Kwon, Mi-Na Kim

**Affiliations:** ^1^QuantaMatrix Inc., Seoul, South Korea; ^2^Department of Laboratory Medicine University of Ulsan College of Medicine and Asan Medical Center, Seoul, South Korea; ^3^Veterans Health Service Medical Center, Seoul, South Korea; ^4^Department of Electrical Engineering and Computer Science, Seoul National University, Seoul, South Korea

**Keywords:** *Mycobacterium tuberculosis*, drug susceptibility testing, Lowenstein-Jensen media, clinical validation, rifampin

## Abstract

There is a high demand for novel approaches to counter the various challenges of conventional drug susceptibility testing (DST) for tuberculosis, the most prevalent infectious disease with significant global mortality. The QMAC-DST system was recently developed for rapid DST using image technology to track the growth of single cells of *Mycobacterium tuberculosis* (MTB). The purpose of this study was to clinically validate the QMAC-DST system compared to conventional DST. In total, 178 MTB isolates recovered from clinical specimens in Asan Medical Center in 2016 were tested by both QMAC-DST and absolute concentration methods using Lowenstein-Jensen media (LJ-DST). Among the isolates, 156 were subjected to DST using BACTEC MGIT 960 SIRE kits (BD, Sparks, MD, United States) (MGIT-DST). The susceptibility/resistance results obtained by QMAC-DST were read against 13 drugs after 7 days of incubation and compared with those of LJ-DST. Based on the gold standard LJ-DST, the agreement rates of QMAC-DST for all drugs were 97.8%, 97.9%, and 97.8% among susceptible, resistant, and total isolates, respectively, while the overall agreement of MGIT-DST tested for 156 isolates against first-line drugs was 95.5%. QMAC-DST showed the highest major error of 6.4% for rifampin, however, it could be corrected by a revised threshold of growth since false-resistant isolates showed grew only half than the true-resistant isolates. The rapid and accurate performance of QMAC-DST warrants ideal phenotypic DST for a wide range of first-line and second-line drugs.

## Introduction

Tuberculosis is a major global health problem, and emergence of multidrug-resistant *Mycobacterium tuberculosis* (MDR-TB) and extensively drug-resistant *M. tuberculosis* TB (XDR-TB) is a threat to public health efforts to control tuberculosis. Therefore, a rapid drug susceptibility testing (DST) is recommended to detect MDR-TB and XDR-TB by Centers for Disease Control and Prevention [CDC] (2006) and World Health Organization [WHO] (2015). Currently, molecular methods to detect drug-resistant MTB are widely available in clinical laboratories due to rapid and robust performance. However, they are only complementary to phenotypic DST, because it is limited to detecting resistance of a few primary drugs whose molecular mechanisms have been well established (Boehme et al., 2010, 2011; Hanrahan et al., 2012). Two reliable phenotypic DST methods based on Lowenstein-Jensen agar medium (LJ-DST) or liquid medium such as the BACTEC MGIT 960 (BD Biosciences, San Jose, CA, United States) have been endorsed by the World Health Organization [WHO] (2015) (Rodrigues et al., 2008; Boehme et al., 2010, 2011; Hanrahan et al., 2012). LJ-DST is the gold standard method for most anti-tuberculosis drugs, but the average turnaround time is 4 weeks. The MGIT-based DST (MGIT-DST) method provides a 7-day turnaround time, which is much faster than LJ-DST (Kim, 2005), but it is labor-intensive and standardized only for streptomycin, isoniazid, rifampin, and ethambutol (SIRE). QMAC-DST (QuantaMatrix, Seoul, Korea) was developed based on microfluidic chip technology to monitor the microscopic growth of MTB colonies (Choi et al., 2016). It has high flexibility in the types and concentrations of drugs tested, no inoculum effects within the wide range of 10^3^–10^8^ cells/mL, excellent reproducibility when using lyophilized drugs, and a rapid turnaround time within 7 days (Jung et al., 2018). For commercialization, a QMAC-DST panel has adapted critical concentrations (CCs) with automatic reading through quantification of microscopic growth, unlike the original design (Jung et al., 2018; Table 1). The purpose of this study was to compare performance of the QMAC-DST system against the two WHO-endorsed DST methods, LJ-DST and MGIT-DST.

**Table 1:** Comparison of three drug susceptibility testing using Lowenstein-Jensen medium, QMAC-DST, and BACTEC MGIT 960.

Drugs	LJ			QMAC				MGIT^a^			
	Concentration	No. of isolates (%)		Critical concentration	No. of isolates (% concordance^b^)			Concentration	No. of isolates (% concordance^b^)		
		S	R		S	R	Total		S	R	Total
Isoniazid	0.2	138 (77.5)	40 (22.5)	0.1	136 (98.6)	40 (100.0)	176 (98.9)	0.1	113 (95.8)	34 (89.5)	147 (94.2)
Rifampin	40	157 (88.2)	21 (11.8)	1	147 (93.6)	21 (100.0)	168 (94.4)	1	136 (99.3)	16 (84.2)	152 (97.4)
Ethambutol	2	167 (93.8)	11 (6.2)	5	166 (99.4)	10 (90.9)	176 (99.0)	5	143 (98.0)	3 (30.0)	146 (93.6)
Streptomycin	10	162 (91.0)	16 (9.0)	2	156 (96.3)	16 (100.0)	172 (96.6)	1	139 (97.9)	12 (85.7)	151 (96.8)
Amikacin	30	178 (100.0)	0 (0.0)	2	178 (100.0)	0 (NA)	178 (100.0)				
Ofloxacin	2	169 (94.9)	9 (5.1)	2	169 (100.0)	9 (100.0)	178 (100.0)				
Moxifloxacin	2	172 (69.6)	6 (3.4)	0.5	169 (98.3)	6 (100.0)	175 (98.3)				
Levofloxacin	2	169 (94.9)	9 (5.1)	1.5	169 (100.0)	9 (100.0)	178 (100.0)				
Kanamycin	30	178 (100.0)	0 (0.0)	2.5	177 (99.4)	0 (NA)	177 (99.4)				
Rifabutin	20	161 (90.5)	17 (9.6)	0.5	157 (97.5)	16 (94.1)	173 (97.2)				
Para-amino salicylic acid	1	174 (97.8)	4 (2.3)	4	168 (96.6)	3 (75.0)	171 (96.1)				
Capreomycin	40	178 (100.0)	0 (0.0)	2.5	176 (98.9)	0 (NA)	176 (98.9)				
Ethionamide	40	167 (93.8)	11 (6.2)	5	154 (92.2)	11 (100.0)	165 (92.7)				
Total		2170 (93.8)	144 (6.2)		2122 (97.8)	141 (97.9)	2263 (97.8)		531 (97.8)	65 (80.3)	596 (95.5)

*a, MGIT DST was performed for 156 of 178 clinical isolates; R, Resistant; S, Susceptible, b, % concordance with LJ. LJ, drug susceptibility testing using Lowenstein-Jensen medium; QMAC, drug susceptibility testing using QMAC-DST; MGIT, drug susceptibility testing using BACTEC MGIT 960; NA, not available due to no resistant isolates.*

## Materials and Methods

### Bacterial Isolates

Clinical MTB isolates were consecutively collected at Asan Medical Center for 12 months from January to December 2015 and stored at 4 ± 2°C until further testing. All isolates used in this study were identified as MTB using the AdvanSure^TM^ TB/NTM real-time PCR (LG Chem, Seoul, Korea) and were submitted to the reference DST, which were analyzed by the Korea Institute of Tuberculosis (Osong, Korea). Up to 200 isolates with enrichment of drug-resistant isolates were collected for the study. All drug-resistant isolates were included, and the remaining isolates were cultured with the susceptible isolates. The study isolates were subcultured on L-J media to confirm viability and purity for this study. This study was approved by the Institutional Review Board of Asan Medical Center (2015-1346). In addition, written consent was waived for the patient from whom the isolate was derived, per the guideline of the *in vitro* test method validation protocol with anonymization.

### QMAC-DST

**FIGURE 1:**
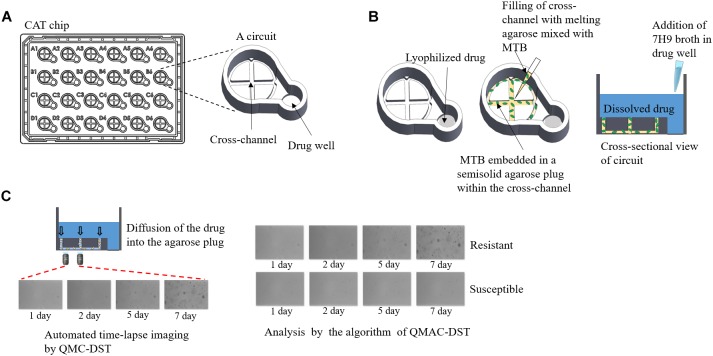
QMAC-DST system for automated time-lapse imaging of CAT chips using image-processing software analysis. **(A)** Schematic representation of a CAT chip with 24 integrated reaction circuits, which is composed of a cross-channel for filling agarose-embedded *Mycobacterium tuberculosis* cells, and a well containing each lyophilized anti-tuberculosis drug. **(B)** After adding 7H9 broth containing 10% OADC, the 0.5% agarose plug filling the cross-channel allowed the dissolved drug to immediately reach the target concentration and make the drugs contact with the embedded *Mycobacterium tuberculosis* cells within a circuit. **(C)** For each circuit, two fields were taken for time-lapse images at fixed positions near the center of the left arm of the cross-channel on days 1, 2, 5, and 7 of incubation. Cumulative colony growth per each field was measured to determine resistant (cell growth) or susceptible (no growth) cells in the circuit using image analysis software.

In total, 178 clinical *M. tuberculosis* isolates were subjected to QMAC-DST against 13 antimicrobials. The reference isolate, *H37Rv* (ATCC 27294), was included in each test run as a control. Phosphate-buffered saline (PBS, pH 6.8) was used to suspend cells from colonies grown on L-J media to prepare a cloudy suspension. Agarose (900 μL, 0.5%; Seakem^®^ LE agarose, Lonza, Rockland, ME, United States) solution was prepared by microwaving and placing in a heat block at 37°C. The cell suspension was mixed with agarose solution at a 1:3 volume ratio after cooling for 3 min (Figure 1; Jung et al., 2018).

All 13 antimicrobials tested, including isoniazid, rifampin, streptomycin, ethambutol, amikacin, capreomycin, ethionamide, kanamycin, levofloxacin, moxifloxacin, ofloxacin, *para*-aminosalicylic acid (PAS), and rifabutin, were purchased from Sigma-Aldrich (St. Louis, MO, United States). The liquid culture medium was Middlebrook 7H9 broth (BBL, BD Biosciences, Baltimore, MD, United States) supplemented with 10% oleic acid, albumin, dextrose, and catalase (BD Biosciences). The Cross Agarose channel for Tuberculosis (CAT) chip was composed of the CCs of each drug. The CCs were based on the Middlebrook 7H9 broth, which were set up for the MGIT 960 system (World Health Organization [WHO], 2009), except streptomycin, amikacin, and PAS, which were adjusted according to a previous study (Jung et al., 2018). The CCs were 0.1, 1.0, 2.0, 5.0, 2.0, 2.5, 2.5, 1.5, 0.5, 2.0, 0.5, 4.0, and 5.0 μg/mL for isoniazid, rifampin, streptomycin, ethambutol, amikacin, capreomycin, kanamycin, levofloxacin, moxifloxacin, ofloxacin, *para*-aminosalicylic acid (PAS), rifabutin, and ethionamide, respectively (Woodley, 1986; Kam et al., 2010; Jung et al., 2018). MTB cell suspension was inoculated as previously described (Jung et al., 2018). The CAT chip was incubated at 37°C, and time-lapsed images were automatically captured on 1, 2, 5, and 7 days of incubation using QDST^TM^ (QuantaMatrix). Colony growth of single cells was monitored each day on a 20× field with an inverted optical microscope, which was always aligned to the same locus by a focus mark engraved on the bottom of the chip (Figure 1; Choi et al., 2014, 2016). The binarization value of the images captured from the fields was calculated as the sum of the black area (cumulative area of colonies) after black and white conversion (binarization). The binarization numbers of each drug-isolate combination were interpreted as susceptibility (S) and resistance (R) using the threshold setting of the QMAC-DST program. For each well, interpretation of susceptibility was duplicated with readings of two separate fields and accepted when both interpretations were matched. The results were compared to those of LJ-DST conducted by the Korea Institute of Tuberculosis. Categorical agreement and error rates were obtained for each drug-isolate combination (Clinical Laboratory Standards Institute [CLSI], 2016). To resolve mismatches between each method, all representative isolates were subjected to retesting and determination of resistant mutations.

### Mycobacteria Growth Indicator Tube (MGIT) Drug Susceptibility Testing

MGIT-DST was performed with the same set of isolates a few months after completion of QMAC-DST using the BACTEC MGIT 960 SIRE kit, according to the manufacturer’s instructions. Briefly, 800 μL of OADC supplement and 100 μL of drugs were added to each MGIT tube. A few colonies on the L-J medium were picked up and suspended in PBS by vortexing to prepare a homogeneous suspension of McFarland 0.5. From this suspension, a 1:5 dilution was prepared using PBS, of which 500 μL samples were inoculated into each drug-containing tube and a growth control tube (Siddiqi et al., 2012; Adami et al., 2017). The drugs were used at the final concentrations recommended by the manufacturer: 1.0 μg/mL streptomycin, 0.1 μg/mL isoniazid, 1.0 μg/mL rifampin, and 5.0 μg/mL ethambutol (Piersimoni et al., 2006). MGIT-DST data were available for 156 isolates, excluding those that were contaminated or failed to grow. The major error (ME), very major error (vME), and agreement rate among the three methods (LJ-DST, MGIT-DST, QMAC-DST) were calculated for each drug (Table 3; Clinical Laboratory Standards Institute [CLSI], 2016).

### Resolution of Discrepancies With Molecular Diagnostic Method

For isolates showing discrepant results, a QMAP Dual-ID (Quantamatrix, Seoul, Republic of Korea) was performed to detect rifampin-resistant mutations. Five wild-type probes representing the wild-type sequence of 81bp hot-spot region (codon 509-534) and five mutant-type probes (513CCA, 516TAC, 516GTC, 526TAC, 531TTG) were used in this study (Wang et al., 2017c).

PCR was performed using 10 μL of Taq HS Perfect Mix (TaKaRa Bio INC., Kusatsu, Shiga, Japan), 2 μL of biotinylated primer mixture, 2–3 μL of sample DNA, and distilled water to a final volume of 20 μL. The PCR thermal cycling conditions were as follows: pre-denaturation for 5 min at 94°C, followed by 40 cycles of 30 s at 94°C, 30 s at 65°C, and final extension of 10 min at 72°C. Biotinylated PCR products were denatured at 25°C for 5 min in denaturation solution. Add 45 μL of provided hybridization solution and probe-coupled microdisks in the provided glass MatriPlate (Brooks, Chelmsford, MA, United States). Transfer 1200 rpm for 30 min. The microdisks were washed three times with 100 μL of washing buffer, incubated with 50 μL of staining buffer (Streptavidin R-phycoerythrin conjugate, ProZyme, San Leandro, CA, United States) at 35°C for 10 min, and washed three times. The results were detected as median fluorescence intensity values using fluorescence microscopy images in the QMAP2.0 Scanner (QuantaMatrix) (Wang et al., 2017a,b,c).

## Results

### Comparison of QMAC-DST and MGIT-DST With LJ-DST

The concordance results of QMAC-DST and MGIT-DST are shown in Table 1. The overall agreement rate of QMAC-DST to LJ-DST for the 13 drugs was 97.8% and that of MGIT-DST for first-line drugs was 95.5%. All drugs showed an agreement rate greater than 90%, except PAS. A vME of QMAC-DST was 25% among the PAS-resistant isolates because there was one mismatch found among four resistant isolates.

### Comparison of Three Methods for Primary Drugs

The discrepant results for primary drugs between each method are shown in Table 2. Rifampin showed the lowest overall agreement rate between QMAC-DST and the reference method.

**Table 2:** Discordant results among three drug susceptibility testing using QMAC-DST, Lowenstein-Jensen medium, and BACTEC MGIT 960 for first-line drugs.

Isolate No.	INH			Isolate No.	RIF			Isolate No.	EMB			Isolate No.	SM		
	QMAC	LJ	MGIT		QMAC	LJ	MGIT		QMAC	LJ	MGIT		QMAC	LJ	MGIT
25	S	S	***R***	36	***R***	S	S	30	S	S	***R***	54	***R***	S	S
30	R	***S***	R	43	***R***	S	S	94	R	R	***S***	102	***R***	S	S
35	S	S	***R***	75	***R***	S	S	109	R	R	***S***	115	***R***	S	S
87	S	S	***R***	76	***R***	S	S	117	S	S	***R***	121	R	R	***S***
106	***R***	S	S	115	***R***	S	S	123	***R***	S	S	123	***R***	S	S
128	S	S	***R***	128	S	S	***R***	141	R	R	***S***	163	S	S	***R***
R10	R	R	***S***	141	R	R	***S***	148	S	S	***R***	R9	R	***S***	R
R13	R	R	***S***	146	R	R	***S***	R11	R	R	***S***	R18	R	R	***S***
R17	R	R	***S***	153	***R***	S	S	R12	R	R	***S***	R21	R	***S***	R
R18	R	R	***S***	155	***R***	S	S	R15	R	R	***S***				
				163	***R***	S	S	R20	R	R	***S***				
				R4	***R***	S	S								
				R10	***R***	S	S								
				R11	R	R	***S***								

*Bold italic letters represent discordant results. LJ, drug susceptibility testing using Lowenstein-Jensen medium; QMAC, drug susceptibility testing using QMAC-DST; MGIT, drug susceptibility testing using BACTEC MGIT 960; INH, Isoniazid; RIF, Rifampin; EMB, Ethambutol; SM, Streptomycin; R, Resistant; S, Susceptible; DST, drug susceptibility testing; MGIT, Mycobacteria growth indicator tube.*

Compared to LJ-DST, the ME and vME rates and overall agreement rates of QMAC-DST and MGIT DST were calculated (Table 3). The QMAC-DST showed ME in 2 (1.7%) for isoniazid, 10 (7.3%) for rifampin, 1 (0.7%) for ethambutol, and 6 (4.2%) for streptomycin, which resulted in total ME of 3.5% but no vME. Therefore, QMAC-DST showed the lowest agreement for rifampin at 93.6%. MGIT-DST showed a low ME of 2.2%, but vME was observed in 3 (15.8%) for rifampin, 7 (70%) for ethambutol, and 2 (14.3%) for streptomycin, which resulted in a 19.8% total vME (Table 3). Among four tested drugs, MGIT-DST showed the lowest agreement for ethambutol at 93.6% due to the occurrence of ME in 3 (2.1%) and vME in 7 (70.0%). The concordance rates of QMAC-DST to MGIT-DST were 94.2% for isoniazid, 91.0% for rifampin, 93.0% for ethambutol, and 95.5% for streptomycin. The overall agreement rates between methods were as follows: 95.5% (MGIT vs. L-J method), 97% (QMAC vs. L-J method), and 93.4% (QMAC vs. MGIT).

**Table 3:** Very major error, major error, and categorical agreement of the drug susceptibility testing using QMAC-DST and BACTEC MGIT 960 compared to drug susceptibility testing using Lowenstein-Jensen medium.

Drugs	LJ		QMAC			MGIT		
	No. of susceptible isolates	No. of resistant Isolates	No. (%) of ME	No. (%) of vME	No. (%) of agreement^a^	No. (%) of ME	No. (%) of vME	No. (%) of agreement^a^
Isoniazid	118	38	2 (1.7)	0 (0.0)	154 (98.7)	5 (4.2)	4 (10.5)	147 (94.2)
Rifampin	137	19	10 (7.3)	0 (0.0)	146 (93.6)	1 (0.7)	3 (15.8)	152 (97.4)
Ethambutol	146	10	1 (0.7)	0 (0.0)	155 (99.4)	3 (2.1)	7 (70.0)	146 (93.6)
Streptomycin	142	14	6 (4.2)	0 (0.0)	150 (96.2)	3 (2.1)	2 (14.3)	151 (96.8)
Total	543	81	19 (3.5)	0 (0.0)	605 (97.0)	12 (2.2)	16 (19.8)	596 (95.5)

*INH, Isoniazid; RIF, Rifampin; EMB, Ethambutol; SM, Streptomycin; R, Resistant; S, Susceptible; DST, drug susceptibility testing; MGIT, Mycobacteria growth indicator tube. a. No.(%) agreement was No.(%) of isolates showing concordant categorical agreement with drug susceptibility testing using Lowenstein-Jensen medium.*

### Analysis of ME Cases of Rifampin in QMAC-DST

**FIGURE 2:**
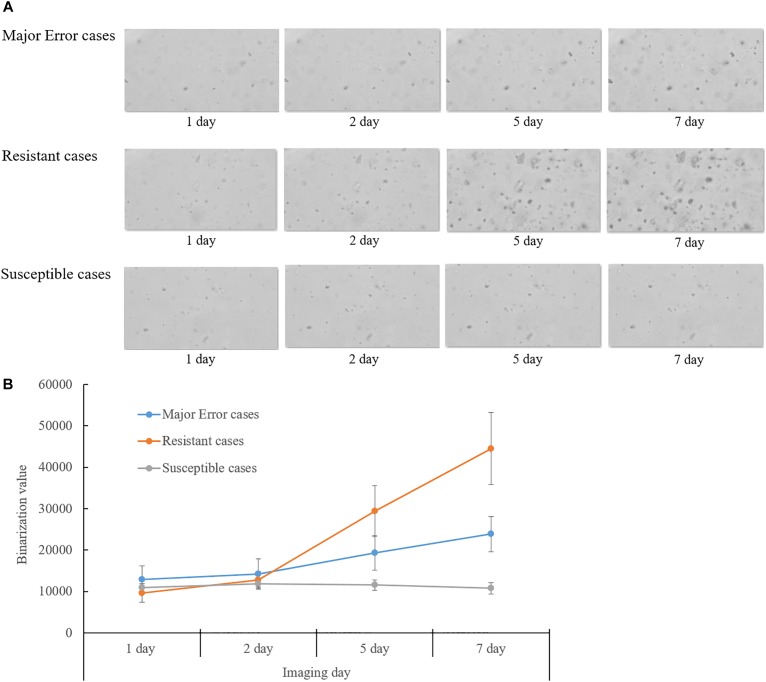
Analysis of QMAC-DST images of isolates showing major error of rifampin DST compared to resistant and susceptible isolates. **(A)** Colony growth of major error cases was greater than susceptible cases, but clearly lesser than resistant cases. **(B)** The binarization value denoted cumulative area of colonies. The value of the major error cases was intermediate between resistant and susceptible cases at day five and seven of incubation.

Eight of ten isolates showing ME in DST for rifampin were confirmed as resistant mutations by the QMAP Dual ID method, which were all resistant in both LJ-DST and MGIT-DST (Table 4). To investigate rifampin’s mechanism of ME, we analyzed images of ME cases compared to isolates that were correctly interpreted to be susceptible or resistant (Figure 2A). Colony growth in these ME cases was less than those for resistant cases but clearly more than those for true susceptible results. The binarization value denoting cumulative area of colonies of these ME cases showed intermediate values between the susceptible and resistant reads, which were only half of the resistant reads in average (Figure 2B).

**Table 4:** Agreement of drug susceptibility testing using QMAC-DST compared to drug susceptibility testing using BACTEC MGIT 960.

Drugs	MGIT		No. (%) of QMAC-R/MGIT-S isolates	No. (%) of QMAC-S/MGIT-R isolates	No. (%) of agreement
	No. of susceptible isolates	No. of resistant Isolates			
Isoniazid	117	39	5 (4.3)	4 (10.3)	147 (94.2)
Rifampin	139	17	13 (9.4)	1 (5.9)	142 (91.0)
Ethambutol	150	6	8 (5.3)	3 (50.0)	145 (93.0)
Streptomycin	141	15	6 (4.3)	1 (6.7)	149 (95.5)
Total	547	77	32 (5.9)	9 (11.7)	583 (93.4)

*INH, Isoniazid; RIF, Rifampin; EMB, Ethambutol; SM, Streptomycin; QMAC-R/MGIT-S, isolates that were susceptible to drug susceptibility testing using BACTEC MGIT 960-DST but resistant to drug susceptibility testing using QMAC-DST; QMAC-S/MGIT-R, isolates that were resistant to drug susceptibility testing using BACTEC MGIT 960-DST but susceptible to drug susceptibility testing using QMAC-DST.*

## Discussion

In this study, QMAC-DST showed a high agreement rate of 97.8% compared to the gold standard method. All drugs except PAS showed a concordance rate of more than 90% in both susceptible and resistant isolates. The microfluidic chip technique applied in QMAC-DST is efficient and highly accommodating in terms of speed and choice of drugs tested because it has adapted the same technology as the established rapid automated antimicrobial susceptibility testing systems for bacterial pathogens, QMAC-dRAST (QuantaMatrix) (Kim et al., 2018). Therefore, compared with other rapid culture-based methods like MGIT, QMAC-DST had an advantage of high throughput, less labor-intensive procedures, and wide availability of various first- and second-line drugs in a single run, similar to QMAC-dRAST. Several phenotypic DST techniques other than MGIT-DST have been established for a rapid turnaround time. Sensititre MYCOTB MIC plate (Trek Diagnostic Systems, Cleveland, OH. United States), microscopic observation drug susceptibility testing method, colorimetric redox DST method (Woods et al., 2015), and phage amplification technology (Albay et al., 2003) are currently available in clinical laboratories. However, they are often limited to first-line drugs, labor intensive, low throughput, or have poor safety issues. Sensititre MYCOTB using 96-well microtiter plates has an advantage to measure real MIC and cover first and second-line drugs as QMAC-DST, but it requires a 3-week incubation for the final read (Lee et al., 2019). Moreover, the high agreement rate to the reference method signifies superior performance combined with its efficiency, which makes QMAC-DST an ideal phenotypic DST system in clinical laboratory, especially in high TB-prevalent countries such as Korea.

No vME found in QMAC-DST showed a high advantage in diagnostic performance. For PAS, showing outliers of ME requires further evaluation with more PAS-resistant isolates because only four resistant isolates were included and one of those was missed in this study. In comparison, MGIT-DST showed lower agreement than QMAC-DST and significant vME. The overall agreement rate of MGIT-DST to LJ-DST was 95.5%, which was slightly lower than previously reported values of 96–97% (Tukvadze et al., 2012; Lawson et al., 2013). This lower agreement rate may be due to the lower resistance rates of the tested isolates in this study compared to those in the previous study. Because this study evaluated only 10 ethambutol-resistant isolates, the vME of MGIT-DST reached 70% when seven isolates were missed by MGIT-DST. Ethambutol resistance is difficult to detect accurately using any phenotypic method because the minimum inhibitory concentration ranges of ethambutol-susceptible and ethambutol-resistant isolates are close to each other. Therefore, the WHO-recommended reference methods are based on various concentrations in the range of 2.0–7.5 μg/mL (Rüsch-Gerdes et al., 1999; Said et al., 2012). In a meta-analysis, the pooled mean based on a high ethambutol concentration of 5 μg/mL as in this study showed the highest agreement with molecular DST (Cheng et al., 2014). In this study, both MGIT-DST and QMAC-DST used 5 μg/mL of ethambutol. In contrast, LJ-DST used 2 μg/mL of ethambutol. Therefore, the reason for the large difference between MGIT-DST and QMAC-DST remains unclear. Comparison of the MGIT-DST and LJ-DST revealed that all first-line drugs showed high concordance rates of greater than 95% in susceptible isolates, but relatively low concordance rates in resistant isolates. The vME of MGIT-DST for isoniazid and rifampin was as high as 10.5% and 15.8%, respectively. Because these two drugs showed very low vME with MGIT-DST in previous studies (Piersimoni et al., 2006; Hwang et al., 2014; Zhao et al., 2014), the relatively small number of resistant isolates was assumed as the only cause of the high vME (Said et al., 2012; Zhao et al., 2014). The higher correlation of QMAC-DST with LJ-DST than MGIT-DST could be explained by its adaptation to the same critical concentrations as LJ-DST. Because vME is the most critical error, this low vME of QMAC-DST warrants diagnostic use.

Comparison of QMAC-DST and LJ-DST revealed high concordance rates of greater than 95%, except for rifampin, in the susceptible isolates and 100%, except for ethambutol, rifabutin, and PAS, in the resistant isolates. There was a large number of the false resistant readings in the rifampin DST. Image analysis indicated that resistance was overcalled due to erroneous reading of growth in rifampin wells. Rifampin is a surrogate marker for detecting MDR when used as a resistant and primary drug for tuberculosis. Therefore, the rifampin DST is critical (Pang et al., 2014), especially in Korea, in which the recent prevalence of rifampin resistance is 5.7% (Lee et al., 2015). Because QMAC-DST results were obtained by observing the growth of single cells using microscopic techniques, threshold settings to discriminate growth may be difficult when only a part of a cell grows as a natural variation of each isolate (Ahmad et al., 2016). However, most of false resistance was found in rifampin wells, and sub-inhibitory effect of rifampin was presumed to play a role. Rifampin is a bactericidal drug that stops protein synthesis by inhibiting DNA-dependent RNA polymerase, however, rifampin also could activate transcription of the quorum-sensing promoter gene at sub-inhibitory concentrations (Acar and Goldstein, 1996). Moreover, the patterns of activation differed in liquid compared to solid media (Goh et al., 2002). Therefore, the effects of conditions, such as agar-embedded bacteria immersed in liquid medium (i.e., QMAC), on growth characteristics of MTB remain unclear. Hence, the breakpoint of QMAC-DST has been optimized further for rifampin based on this study, and thus all MEs have been successfully adjusted.

A limitation of this study was that it did not contain a sufficient number of resistant isolates for all drugs. The resistance to second-line drugs is still very low, mostly less than 3% in Korea (Lee et al., 2015). Another limitation is that pyrazinamide (PZA), a first-line drug, was not included in the QMAC-DST panel. Because (PZA) requires different pH values, unlike other drugs, it is currently under development to add to the QMAC-DST panel.

## Conclusion

In conclusion, QMAC-DST is a rapid and accurate alternative to conventional DST. Additionally, QMAC-DST is highly valuable in cases of treatment failure or contact history for MDR-TB patients because it can test various first- and second-line drugs. This fully automated, high-throughput DST system is particularly suitable for high-capacity laboratories.

## Author Contributions

M-NK and SHK equally contributed to whole processing of this study as a corresponding authors. SL led the experiments and wrote this manuscript. DC, YC, EJ, SYK, HK, HJK, JC, HS, and BJ contributed to performance of the experiments and data analysis. GK contributed to the sequencing data analysis. E-GK contributed to the design and production of the DST chip.

## Conflict of Interest Statement

SL, EJ, SYK, HK, HJK, GK, BJ, E-GK and SHK were employed at QuantaMatrix, Inc., which is developing and commercializing the DST technology and has an equity interest in QuantaMatrix, Inc. The remaining authors declare that the research was conducted in the absence of any commercial or financial relationships that could be construed as a potential conflict of interest.

## References

[B1] Acar J. F. Goldstein F. W. (1996). “Disk susceptibility testing,” in *Antibiotics in Laboratory Medicine*, ed. Lorian V. (Baltimore, MD: Williams & Wilkins), 419–420. 10.1016/j.diagmicrobio.2016.10.023

[B2] Adami A. G. Gallo J. F. Pinhata J. M. Martins M. C. Giampaglia C. M. de Oliveira R. S. (2017). Modified protocol for drug susceptibility testing of MGIT cultures of *Mycobacterium tuberculosis* by the MGIT 960. *Diagn. Microbiol. Infect. Dis.* 87 108–111. 10/1016/j.diagmicrobio.2016.10.023 27889251 10.1016/j.diagmicrobio.2016.10.023

[B3] Ahmad S. Mokaddas E. Al-Mutairi N. Eldeen H. S. Mohammadi S. (2016). Discordance across phenotypic and molecular methods for drug susceptibility testing of drug-resistant *Mycobacterium tuberculosis* isolates in a low TB incidence country. *PLoS One* 11:e0153563. 10.1371/journal.pone.0153563 27096759 PMC4838278

[B4] Albay A. Kisa O. Baylan O. Doganci L. (2003). The evaluation of FASTPlaqueTB test for the rapid diagnosis of tuberculosis. *Diagn. Microbiol. Infect. Dis.* 46 211–215. 10.1016/S0732-8893(03)00048-48 12867097

[B5] Boehme C. C. Nabeta P. Hillemann D. Nicol M. P. Shanai S. Krapp F. (2010). Rapid molecular detection of tuberculosis and rifampin resistance. *N. Engl. J. Med.* 363 1005–1015. 10.1056/NEJMoa0907847 20825313 PMC2947799

[B6] Boehme C. C. Nicol M. P. Nabeta P. Michael J. S. Gotuzzo E. Tahirli R. (2011). Feasibility, diagnostic accuracy, and effectiveness of decentralised use of the Xpert MTB/RIF test for diagnosis of tuberculosis and multidrug resistance: a multicentre implementation study. *Lancet* 377 1495–1505. 10.1016/S0140-6736(11)60438-60438 21507477 PMC3085933

[B7] Centers for Disease Control and Prevention [CDC] (2006). Emergence of *Mycobacterium tuberculosis* with extensive resistance to second-line drugs-worldwide. 2000-2004. *MMWR Morb. Mortal. Wkly. Rep.* 55 301–305. 16557213

[B8] Cheng S. Cui Z. Li Y. Hu Z. (2014). Diagnostic accuracy of a molecular drug susceptibility testing method for the antituberculosis drug ethambutol: a systematic review and meta-analysis. *J. Clin. Microbiol.* 52 2913–2924. 10.1128/JCM.00560-514. 24899018 PMC4136177

[B9] Choi J. Kim E. K. Yoo J. Lee M. Lee S. Joo S. (2014). Rapid antimicrobial susceptibility test based on single cell morphological analysis. *Sci. Transl. Med.* 6:174. 10.1126/scitranslmed.3009650 25520395

[B10] Choi J. Yoo J. Kim K. J. Kim E. G. Park K. O. Kim H. (2016). Rapid drug susceptibility test of *Mycobacterium tuberculosis* using microscopic time-lapse imaging in an agarose matrix. *Appl. Microbiol. Biotechnol.* 100 2355–2365. 10.1007/s00253-015-7210-7210 26754815

[B11] Clinical Laboratory Standards Institute [CLSI] (2016). “Approved Guideline M23-A4,” in *Development of In vitro Susceptibility Testing Criteria and Quality Control Parameters*, 4th edn, ed. Matthew A. (Wayne, PA: Clinical Laboratory Standards Institute). 10.1073/pnas.252607699

[B12] Goh E. B. Yim G. Tsui W. McClure J. Surette M. G. Davies J. (2002). Transcriptional modulation of bacterial gene expression by subinhibitory concentrations of antibiotics. *Proc. Natl. Acad. Sci. U.S.A.* 99 17025–17030. 10.1073/pnas.252607699 12482953 PMC139263

[B13] Hanrahan C. F. Dorman S. E. Erasmus L. Koornhof H. Coetzee G. Golub J. E. (2012). The impact of expanded testing for multidrug resistant tuberculosis using genotype MTBDRplus in South Africa: an observational cohort study. *PLoS One* 7:e49898. 10.1371/journal.pone.0049898 23226229 PMC3511489

[B14] Hwang S. M. Hwang K. C. Hong Y. J. Lee H. R. Kim T. S. Park K. U. (2014). Improving antitubercular drug susceptibility testing with liquid media. *Ann. Clin. Lab. Sci.* 44 123–130. 10.1038/s41598-018-26419-z 24795049

[B15] Jung Y. G. Kim H. Lee S. Kim S. Jo E. Kim E. G. (2018). A rapid culture system uninfluenced by an inoculum effect increases reliability and convenience for drug susceptibility testing of *Mycobacterium tuberculosis*. *Sci. Rep.* 8:8651. 10.1038/s41598-018-26419-z 29872060 PMC5988837

[B16] Kam K. M. Sloutsky A. Yip C. W. Bulled N. Seung K. J. Zignol M. (2010). Determination of critical concentrations of second-line anti-tuberculosis drugs with clinical and microbiological relevance. *Int. J. Tuberc. Lung Dis.* 14 282–288. 10.5145/ACM.2018.21.1.12 20132618

[B17] Kim H. Jeong H. Y. Han S. K. Han S. H. Choi J. Jin B. (2018). Clinical evaluation of QMAC-dRAST for direct and rapid antimicrobial susceptibility test with gram-positive cocci from positive blood culture bottles. *Ann. Clin. Microbiol.* 21 12–19. 10.5145/ACM.2018.21.1.12

[B18] Kim S. J. (2005). Drug-susceptibility testing in tuberculosis: methods and reliability of results. *Eur. Respir. J.* 25 564–569. 10.1183/09031936.05.00111304 15738303

[B19] Lawson L. Emenyonu N. Abdurrahman S. T. Lawson J. O. Uzoewulu G. N. Sogaolu O. M. (2013). Comparison of *Mycobacterium tuberculosis* drug susceptibility using solid and liquid culture in Nigeria. *BMC Res. Notes* 6:215. 10.1186/1756-0500-6-215 23721428 PMC3691748

[B20] Lee H. Y. Lee J. Lee Y. S. Kim M. Y. Lee H. K. Lee Y. M. (2015). Drug-resistance pattern of *Mycobacterium tuberculosis* strains from patients with pulmonary and extrapulmonary tuberculosis during 2006 to 2013 in a Korean tertiary medical center. *Korean J. Intern. Med.* 30 325–334. 10.3904/kjim.2015.30.3.325 25995663 PMC4438287

[B21] Lee J. Armstrong D. T. Ssengooba W. Park J. A. Yu Y. Mumbowa F. (2019). Sensititre MYCOTB MIC plate for testing *Mycobacterium tuberculosis* susceptibility to first- and second-line drugs. *Antimicrob. Agents Chemother.* 58 11–18. 10.1128/AAC.01209-1213 24100497 PMC3910714

[B22] Pang Y. Ruan Y. Z. Zhao J. Chen C. Xu C. H. Su W. (2014). Diagnostic dilemma: treatment outcomes of tuberculosis patients with inconsistent rifampicin susceptibility. *Int. J. Tuberc. Lung Dis.* 18 357–362. 10.5588/ijtld.13.0459 24670576

[B23] Piersimoni C. Olivieri A. Benacchio L. Scarparo C. (2006). Current perspectives on drug susceptibility testing of *Mycobacterium tuberculosis* complex: the automated nonradiometric systems. *J. Clin. Microbiol.* 44 20–28. 16390942 10.1128/JCM.44.1.20-28.2006 PMC1351976

[B24] Rodrigues C. Jani J. Shenai S. Thakkar P. Siddiqi S. Mehta A. (2008). Drug susceptibility testing of *Mycobacterium tuberculosis* against second-line drugs using the bactec MGIT 960 system. *Int. J. Tuberc. Lung Dis.* 12 1449–1455. 19017456

[B25] Rüsch-Gerdes S. Domehl C. Nardi G. Gismondo M. R. Welscher H. M. Pfyffer G. E. (1999). Multicenter evaluation of the mycobacteria growth indicator tube for testing susceptibility of *Mycobacterium tuberculosis* to first-line drugs. *J. Clin. Microbiol.* 37 45–48. 10.1186/1471-2334-12-369 9854062 PMC84164

[B26] Said H. M. Kock M. M. Ismail N. A. Baba K. Omar S. V. Osman A. G. (2012). Comparison between the BACTEC MGIT 960 system and the agar proportion method for susceptibility testing of multidrug resistant tuberculosis strains in a high burden setting of South Africa. *BMC Infect. Dis.* 12:369. 10.1186/1471-2334-12-369 23259765 PMC3543708

[B27] Siddiqi S. Ahmed A. Asif S. Behera D. Javaid M. Jyoti A. (2012). Direct drug susceptibility testing of *Mycobacterium tuberculosis* for rapid detection of multidrug resistance using the Bactec MGIT 960 system: a multicenter study. *J. Clin. Microbiol.* 50 435–440. 10.1371/journal.pone.0031563 22162558 PMC3264138

[B28] Tukvadze N. Kempker R. R. Kalandadze I. Kurbatova E. Leonard M. K. Apsindzelashvili R. (2012). Use of a molecular diagnostic test in AFB smear positive tuberculosis suspects greatly reduces time to detection of multidrug resistant tuberculosis. *PLoS One* 7:e31563. 10.1371/journal.pone.0031563 22347495 PMC3276512

[B29] Wang H. Y. Uh Y. Kim S. Lee H. (2017a). Performance of the quantamatrix multiplexed assay platform system for the differentiation and identification of *Mycobacterium* species. *J. Med. Microbiol.* 66 777–787. 10.1099/jmm.0.000495 28604333

[B30] Wang H. Y. Uh Y. Kim S. Lee H. (2017b). Quantamatrix multiplexed assay platform system for direct detection of bacteria and antibiotic resistance determinants in positive blood culture bottles. *Clin. Microbiol. Infect.* 23:333. 10.1016/j.cmi.2016.12.013 27998819

[B31] Wang H. Y. Uh Y. Kim S. Shim T. S. Lee H. (2017c). Evaluation of the quantamatrix multiplexed assay platform system for simultaneous detection of *Mycobacterium tuberculosis* and the rifampicin resistance gene using cultured mycobacteria. *Int. J. Infect. Dis.* 61 107–113. 10.1016/j.ijid.2017.06.008 28627431

[B32] Woodley C. L. (1986). Evaluation of streptomycin and ethambutol concentrations for susceptibility testing of *Mycobacterium tuberculosis* by radiometric and conventional procedures. *J. Clin. Microbiol.* 23 385–386. 3084552 10.1128/jcm.23.2.385-386.1986 PMC268651

[B33] Woods G. L. Lin S. Y. G. Desmond E. P. (2015). “Susceptibility test methods: *Mycobacteria*, *Nocardia* and other Actinomycetes” in *Manual of Clinical Microbiology*, 11th edn, ed. Jorgensen J. H. (Washington, DC: ASM Press).

[B34] World Health Organization [WHO] (2009). Guidelines for surveillance of drug resistance in tuberculosis, 4th edn. Geneva: WHO.

[B35] World Health Organization [WHO] (2015). Global tuberculosis report. Geneva: World Health Organization.

[B36] Zhao P. Fang F. Yu Q. Guo J. Zhang J. H. Qu J. (2014). Evaluation of BACTEC MGIT 960 system for testing susceptibility of *Mycobacterium tuberculosis* to first-line drugs in China. *PLoS One* 9:e99659. 10.1371/journal.pone.0099 25248118 PMC4172428

